# The *H. pylori* CagA Oncoprotein Induces DNA Double Strand Breaks through Fanconi Anemia Pathway Downregulation and Replication Fork Collapse

**DOI:** 10.3390/ijms23031661

**Published:** 2022-01-31

**Authors:** Arun Mouli Kolinjivadi, Haresh Sankar, Ramveer Choudhary, Lavina Sierra Tay, Tuan Zea Tan, Naoko Murata-Kamiya, Dominic Chih-Cheng Voon, Dennis Kappei, Masanori Hatakeyama, Vaidehi Krishnan, Yoshiaki Ito

**Affiliations:** 1 Cancer Science Institute of Singapore, National University of Singapore, Singapore 117599, Singapore; arunkumarkc@instem.res.in (A.M.K.); haresh.shankar23@gmail.com (H.S.); dennis.kappei@nus.edu.sg (D.K.); 2 Center for Chemical Biology & Therapeutics, inStem & NCBS, Bellary Road, Bangalore 560065, India; 3 FIRC Institute of Molecular Oncology (IFOM), 20139 Milan, Italy; ramveerc@gmail.com; 4 Genome Institute of Singapore, Agency for Science, Technology and Research, Singapore 138672, Singapore; taylavinasierra@gmail.com; 5 Genomic and Data Analytics Core (GeDaC), Cancer Science Institute, National University of Singapore, Singapore 117599, Singapore; csittz@nus.edu.sg; 6 Department of Microbiology, Graduate School of Medicine, University of Tokyo, Tokyo 113-0033, Japan; naokokam@m.u-tokyo.ac.jp (N.M.-K.); mhata@m.u-tokyo.ac.jp (M.H.); 7 Institute for Frontier Science Initiative, Kanazawa University, Kanazawa 920-1192, Japan; dvoon@staff.kanazawa-u.ac.jp; 8 Cancer Research Institute, Kanazawa University, Kanazawa 920-1192, Japan; 9 Department of Biochemistry, Yong Loo Lin School of Medicine, National University of Singapore, Singapore 117597, Singapore; 10 NUS Center for Cancer Research, Yong Loo Lin School of Medicine, National University of Singapore, Singapore 117597, Singapore; 11 Cancer and Stem Cell Biology Program, Duke-NUS Graduate Medical School, Singapore 169857, Singapore

**Keywords:** *H. pylori*, DNA replication, CagA, DNA double strand breaks, Fanconi Anemia, γH2AX

## Abstract

The proteins from the Fanconi Anemia (FA) pathway of DNA repair maintain DNA replication fork integrity by preventing the unscheduled degradation of nascent DNA at regions of stalled replication forks. Here, we ask if the bacterial pathogen *H. pylori* exploits the fork stabilisation machinery to generate double stand breaks (DSBs) and genomic instability. Specifically, we study if the *H. pylori* virulence factor CagA generates host genomic DSBs through replication fork destabilisation and collapse. An inducible gastric cancer model was used to examine global CagA-dependent transcriptomic and proteomic alterations, using RNA sequencing and SILAC-based mass spectrometry, respectively. The transcriptional alterations were confirmed in gastric cancer cell lines infected with *H. pylori.* Functional analysis was performed using chromatin fractionation, pulsed-field gel electrophoresis (PFGE), and single molecule DNA replication/repair fiber assays. We found a core set of 31 DNA repair factors including the FA genes FANCI, FANCD2, BRCA1, and BRCA2 that were downregulated following CagA expression. *H. pylori* infection of gastric cancer cell lines showed downregulation of the aforementioned FA genes in a CagA-dependent manner. Consistent with FA pathway downregulation, chromatin purification studies revealed impaired levels of Rad51 but higher recruitment of the nuclease MRE11 on the chromatin of CagA-expressing cells, suggesting impaired fork protection. In line with the above data, fibre assays revealed higher fork degradation, lower fork speed, daughter strands gap accumulation, and impaired re-start of replication forks in the presence of CagA, indicating compromised genome stability. By downregulating the expression of key DNA repair genes such as FANCI, FANCD2, BRCA1, and BRCA2, *H. pylori* CagA compromises host replication fork stability and induces DNA DSBs through fork collapse. These data unveil an intriguing example of a bacterial virulence factor that induces genomic instability by interfering with the host replication fork stabilisation machinery.

## 1. Introduction

The *H. pylori* pathogen colonises the stomach of over half the world’s population and has evolved to survive the hostile gastric environment and promote neoplastic transformation [1]. The pathogenicity of *H. pylori* is dependent on the cag pathogenicity island (cag-PAI), a ~40 Kb segment region within the bacterial genome that encodes the type IV secretion system (T4SS) and the virulence factor, Cytotoxin-associated gene A (CagA) [2]. Through T4SS binding to host α5β1 integrin receptors, CagA is injected into the host where it undergoes phosphorylation by the Src kinases [2]. CagA then physically interacts and disrupts major cell signalling networks regulated by SHP2, c-Met, Ras-Erk, Wnt, and PAR1 kinase and activates pro-oncogenic properties such as inflammation, proliferation, epithelial to mesenchymal transition, and genomic instability [3,4,5]. 

The link between *H. pylori* infection and genomic instability has been examined in previous studies [6,7,8,9,10,11]. For example, higher levels of oxidative stress and DNA damage were shown to accumulate in *H. pylori*-infected cultured cells and gastric mucosal biopsies of infected patients [3]. As one of the possible mechanisms, an elevated expression of the pro-oxidant genes, spermine oxidase (SMO) and NFkB/inducible nitric oxide synthase (iNOS), cytokine and chemokines were shown to increase oxidative stress and DNA damage. However, the addition of reactive oxygen species (ROS) sequestrating agents did not completely ameliorate DNA damage, suggesting additional mechanisms for DSB formation. As an alternative mechanism of DSB induction, the transcription factor NFkB was shown to form a complex with the nucleotide excision repair nucleases XPG and XPF, which in turn programmed DSBs at target gene promoters after *H. pylori* infection [12]. However, it remains unknown if CagA expression induces DSBs through the NER pathway. More recently, CagA-dependent inhibition of Par1b, a member of the PAR1 serine/threonine kinase family of proteins was shown to induce CagA-dependent DSBs [3]. Consistently, the re-introduction of ectopic Par1b rescued the DSBs induced by CagA-positive *H. pylori* by preventing BRCAness [8].

As such, the aforementioned studies implied that *H. pylori*-mediated DSBs at the initial stages of carcinogenesis may enable progressive mutational accumulation during the multi-step cascade of gastric cancer. Direct causal evidence for this model was obtained by chromatin immunoprecipitation (CHIP) analysis of *H. pylori* infected cells with the γH2AX (DSB marker) antibody. DNA damage was found to accumulate mainly in the genic and transcribed regions and at chromosomal ends [9]. Interestingly, genomic regions susceptible to *H. pylori*-mediated DNA damage correlated well with the focal amplifications that develop eventually at the stage of gastric cancer. In the same study, transcriptional profiling of *H. pylori* infected cells revealed downregulation of DNA damage response (DDR) factors [9], although the CagA dependence of this phenomenon was not examined. Moreover, it is not clear how exactly DDR downregulation mechanistically translates into DSBs in the host genome.

Here, we use both CagA-inducible and infection models and performed gene expression profiling, proteomic analysis, chromatin fractionation, and replication fork protection/re-start assays. We show that CagA downregulates major DNA repair factors of the FA pathway such as FANCI, FANCD2, BRCA1, and BRCA2, which eventually culminates in impaired replication fork stability and DSB induction. Thus, our data identify a novel mechanism of *H. pylori*-induced genomic instability.

## 2. Results

### 2.1. CagA Induces Host Double Strand Breaks and Downregulates FA Repair Factors

**Figure 1:**
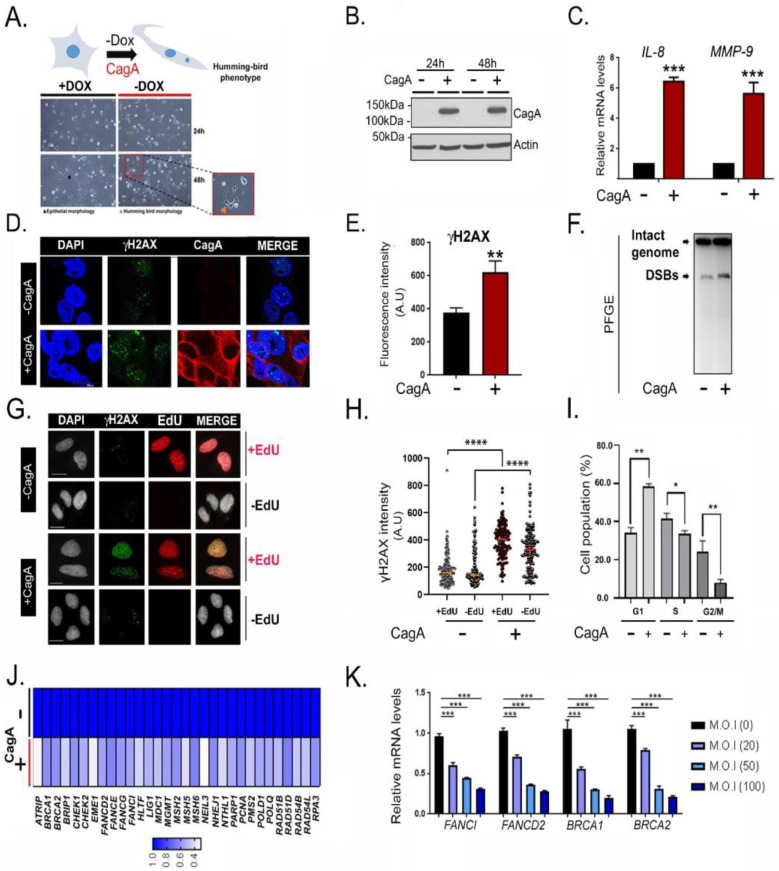
The *H. pylori* oncoprotein CagA induces host DSBs and downregulates FA repair factors. (**A**) Dox-inducible MKN28−CagA cells were either left untreated (−CagA) or subjected to doxycycline withdrawal (+CagA) for 24 h or 48 h as indicated. Representative images displaying hummingbird morphology in CagA-expressing cells (cartoon-inset); scale bar = 100 μM. (**B**) Immunoblot indicating the expression of HA-tagged CagA at two different time points following doxycycline withdrawal (24 and 48 h). Western blots were probed with anti-HA antibody**.** (**C**) Q-PCR for *IL-8* and *MMP9* was performed 48 h following doxycycline withdrawal. (**D**) Representative immunofluorescence images stained for DAPI, HA, and γH2AX. (**E**) Quantification of nuclear γH2AX fluorescence in the presence or absence of CagA using the operetta high-high content imaging system (methods). (**F**) Pulsed-Field Gel Electrophoresis image showing DNA DSBs in CagA-expressing cells**.** (**G**) Representative immunofluorescence images showing EdU labelling using click-IT reaction and γH2AX staining in the presence and absence of CagA expression. (**H**) Quantification of nuclear γH2AX and EdU in the presence and absence of CagA. A total of at least 200 cells were analysed per experimental condition with three biological replicates *n* = 3. (**I**) MKN28 cells were cultured in presence or absence of Dox for 48 hrs. Cells were then stained with propidium iodide and were subjected to cell cycle analysis using flow cytometry. Percentages of cells in G1 and S and G2 phases are shown. The experiments were performed in triplicate. (**J**). Dox-inducible MKN28−CagA cells were left untreated (−CagA) or subjected to doxycycline withdrawal (+CagA) for 72 h following by RNA-seq analysis. Heatmap showing the status of 31 downregulated DNA repair genes upon CagA expression. (**K**)**.** MKN28 cells were infected with wild-type *H. pylori* for 24 h at the indicated multiplicity of infection (M.O.I). Samples were harvested for Q-PCR analysis and the normalized expression levels of *FANCI, FANCD2*, *BRCA1*, and *BRCA2* are shown (n = 3). Student’s *t*-test was performed for statistical analysis. Graphs show mean ± SD. Asterisks represent significant differences. * *p* < 0.05; ** *p* < 0.01; *** *p* < 0.001; **** *p* < 0.0001.

A doxycycline-inducible CagA model was used to examine the relationship between CagA expression and DSB induction. As described earlier [8,11], upon doxycycline withdrawal, CagA-expressing MKN28 cells exhibited characteristic hummingbird morphology and upregulation of *IL-8* and *MMP-9* (Figure 1A–C and Figure S1A), demonstrating that CagA was functional and recapitulated the known effects of this bacterial protein on gastric epithelial cells. Employing this system, we asked if CagA expression triggers spontaneous DSBs. Antibodies recognizing DSBs (γH2AX-Ser139 and pATM (Ser1981) and DDR signalling read-outs (pSQ/TQ and pCHK2) were used as markers of DNA damage response and repair. CagA expression resulted in a substantial increase in both DSBs and DDR (CagA positive versus CagA negative: γH2AX, *p* = 0.002; pATM, *p* = 5.13E-05, pSQ/TQ, *p =* 1.3E-05; pCHK2, *p =* 0.002) (Figure 1E and Figure S1B–D). Pulsed-field gel electrophoresis (PFGE) independently revealed higher DSB accumulation upon CagA expression (Figure 1F). Moreover, CagA-dependent DSB accumulation was particularly more pronounced in the S phase of cell cycle, as revealed through the co-staining of γH2AX with the S phase marker, 5-ethynyl-2′-deoxyuridine (EdU) (Figure 1G,H). However, consistent with previous reports [8,13], cell cycle analyses suggested that after 48 h of CagA expression, there is a significant proportion of cells accumulated in G1 (Figure 1I). Our results demonstrate that CagA expression was sufficient to trigger genomic DNA DSBs within the host.

To examine the pathways deregulated by CagA in an unbiased manner, we profiled the transcriptome of CagA-expressing cells through RNA sequencing. Differentially expressed genes (DEGs) that were either upregulated (1605 genes) or downregulated (1311 genes) (1.5-fold difference, PPEE < 0.05, and PPDE > 0.95) following CagA expression were computed (Tables S1 and S2). Gene ontology (GO) analysis of DEGs revealed “Ras GTPase binding”, “Cell-cell adhesion”, and “Inflammatory response”, among the highly overexpressed pathways upon CagA induction (Figure S1E, (i)). As reported earlier, *IL-8, MMP9, IL23A*, and *TLR6* were among the most significantly overexpressed genes, confirming the known effects of CagA expression (Table S1). On the other hand, the terms “Cell Cycle”, “mitosis”, and “DNA repair” were among the pathways significantly attenuated by CagA expression (Figure S1E, (i) and Table S2). Importantly, gene-set enrichment analysis (GSEA) further confirmed significant downregulation of DNA repair genes in CagA-expressing cells (*p* < 0.0001, normalised enrichment score (NES) = −2.39) (Figure S1E, (ii)). In summary, a set of 31 DNA repair factors that were downregulated by CagA at least by ~2-fold were identified (Figure 1J). Of the downregulated DNA repair genes, 11 were essential components of the Fanconi Anemia (FA)/homologous recombination (HR) pathway of DNA repair.

The FA/HR pathway is a central genome maintenance network involved in DNA inter-strand crosslink (ICLs) repair [14]. During ICL repair, FANCI and FANCD2 are mono-ubiquitinated and recruited to the sites of DNA damage following which the HR proteins BRCA1 and BRCA2 are engaged to promote RAD51 recruitment and complete HR. As such, the impaired functioning of the FA pathway is unequivocally linked to genomic instability and malignancy [14]. FA genes such as *FANCI, FANCD2, FANCE, FANCG, BRCA1, BRCA2, BRIP1,* and accessory FA factors such as *EME1, RPA3, PMS2*, and *ATRIP* were consistently downregulated by CagA (Figure S1F).

Next, *H. pylori* infection models were used to validate the downregulation of FA factors in a CagA-dependent manner. MKN-28 cells were infected with an increasing multiplicity of infection (M.O.I) of *H. pylori* (M.O.I of 20, 50, and 100) for 24 h and Q-PCR analysis was performed. In agreement with the CagA-induction data, significant transcriptional downregulation of *FANCI*, *FANCD2,* *BRCA1,* and *BRCA2* was observed with increasing M.O.I of *H. pylori* infection (Figure 1K). Then, to examine the CagA-dependence of repair gene downregulation, AGS cells were either non-infected or infected with either wild-type (WT) *H. pylori* strain or the CagA-deleted (ΔcagA) version. *BRCA1, BRCA2, FANCI,* and *FANCD2* were substantially downregulated following WT-*H. pylori* infection, but not upon infection with the ΔcagA *H. pylori* strain (Figure S1G), highlighting the CagA-dependence in their downregulation.

### 2.2. Proteomic Approach Further Revealed a Reduction in Fanconi Anemia Factors 

**Figure 2:**
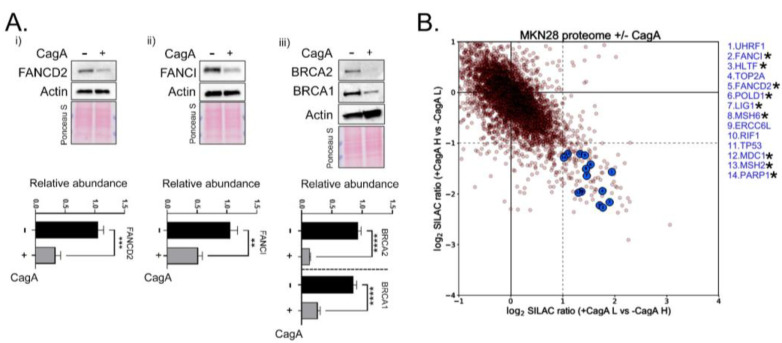
Protein level reduction in Fanconi Anemia and Homologous Recombination Factors (**A**). Dox-inducible MKN28−CagA cells were either left untreated (−CagA) or subjected to doxycycline withdrawal (+CagA) for 60 h and samples were harvested for Western blot and probed for FANCD2 (i), FANCI (ii), BRCA2 (iii), and BRCA1 (iii). Actin was used as the loading control. Below is the quantification of the blots from three independent experiments. Asterisks represent significant differences, * *p* < 0.05; ** *p* < 0.01; *** *p* < 0.001; **** *p* < 0.0001; (**B**) Plots represent proteins that are differentially expressed upon CagA induction (72 h). The cut-offs for significant differential expression was set to log2 (fold change) >1 across the forward (X-axis) and reverse (Y-axis) SILAC-based proteomic quantifications. Blue circles depict DNA repair proteins downregulated by at least 2-fold upon CagA expression across the two experiments. Asterisks are indicated against proteins that were also identified as DEGs following CagA expression. The complete plot is shown in Figure S2A.

Next, to examine the proteomic alterations induced upon CagA expression, we initially confirmed through Western blots that FA factors, FANCI, FANCD2, BRCA1, and BRCA2 were indeed lower in abundance at the protein level (Figure 2A). However, to interrogate the global proteomic changes elicited by CagA expression in an unbiased manner, we performed SILAC (stable isotope labelling by amino acids in cell culture)-based mass spectrometry analysis. The entire list of proteins differentially expressed at the proteomic level upon CagA expression was plotted (Supplementary Materials Figure S2A and Tables S3–S5). While proteomics data are typically less comprehensive compared to RNA-seq, we identified 14 DNA repair factors that had at least 2-fold lower abundance upon CagA expression (Figure 2B). Of these, 9 proteins namely FANCI, FANCD2, MSH2, MSH6, LIG1, HLTF, MDC1, PARP1, and POLD1 had high peptide coverages, were quantified with SILAC ratios in both the forward and reverse experiments, and were shared with the RNA-seq dataset as downregulated genes (proteins marked with asterisk in Figure 2B). Additionally, 5 more DNA repair factors (UHFR1, TOP2A, TP53, XRCC6L, and RIF1) were specifically found to be expressed with at least 2-fold lower levels only upon proteomic quantification, suggesting their misregulation upon CagA expression is post-transcriptional. Of note, higher p53 degradation upon CagA expression has been demonstrated earlier [15], while the other newly identified proteins may have novel roles in CagA-induced genomic instability and can be a valuable resource for *H. pylori*-based studies.

### 2.3. Impairment in Fanconi Anemia Gene Expression Compromises Replication Fork Stability and Induces DSBs in CagA-Expressing Cells

**Figure 3:**
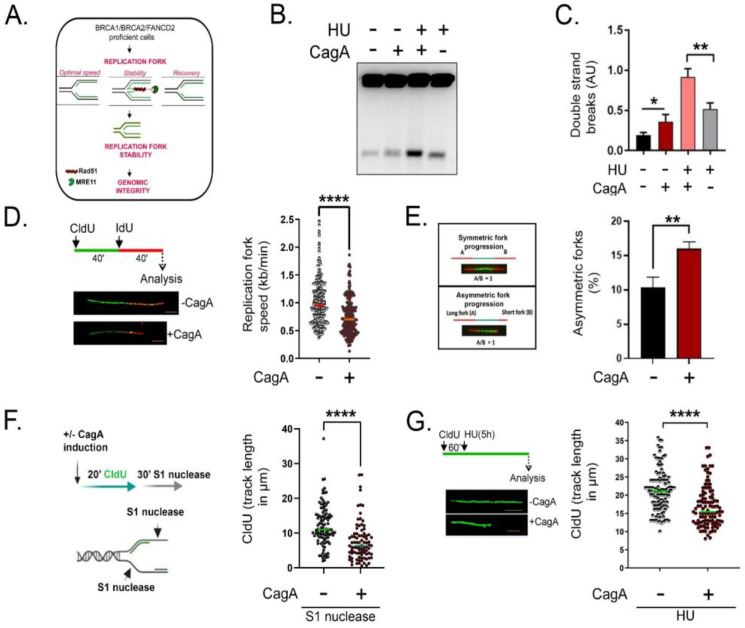
FA genes down-regulation following CagA expression reduces replication fork speed, accumulate single strand gaps, and compromise replication fork stability *(***A**). Model showing the requirement of FA/HR factors for the maintenance of replication fork speed, stability, and re-start concertedly translating into fork stabilisation and genomic integrity. (**B**) Pulsed field gel electrophoresis image showing DNA DSBs in the presence or absence of CagA. Cells were optionally supplemented with HU (0.2 mM HU for 8 h). (**C**) Quantification of Double-Strand Breaks (DSBs) intensity (AU) (**D**) Fork Speed-Fibre Assay Scheme: Cells were exposed sequentially to CldU and IdU for 40 min each. Replication speed was calculated by measuring the CIdU and IdU tract (only connected fibres were considered for the statistical analysis). Scale bar= 5 kb, *p =* < 0.0001*,* Mann–Whitney test, *n* = 250 fibres each condition. The experiments were performed at least three times. (**E**) (Left) Representative image depicting the difference between symmetric and asymmetric replication forks is shown. (Right) Asymmetric forks were measured by dividing the long fork by the short fork. A minimum of 75 fibres were counted for each condition and the percentage was calculated. The experiment was repeated at least thrice to reach similar results. (**F**) S1 nuclease assay scheme—In the presence or absence of CagA, cells were labelled for 20 min with CIdU and subjected to S1 nuclease treatment for 30 min. At least 75 fibres were scored in two independent experiment yielding similar results. (**G**) Fork degradation-Fibre assay scheme: Cells were pulsed labelled with CIdU for 60 min, following which they were exposed to HU (4 mM) for 5 h. Replication fork degradation was calculated at least from 100 fibres for each condition by measuring the median CIdU tract length. The experiment was performed twice and similar results were obtained. Asterisks represent significant differences. * *p* < 0.05; ** *p* < 0.01; **** *p* < 0.0001.

We next asked if the downregulation of FA/HR factors is responsible for DNA DSBs following CagA expression. The FA/HR proteins (BRCA1, BRCA2, and FANCD2) are critical for recognising, repairing, and re-starting stalled replication forks, and defects in this pathway translate into fork destabilisation and DSBs [14,16,17,18]. For example, FA/HR factors promote RAD51 nucleofilament formation and alleviate unscheduled fork degradation by nucleases such as MRE11 [14,16,19]. Moreover, the FA/HR proteins BRCA2 and RAD51 regulate optimal fork speed [20] and lower fork speed is known to elevate replication stress [18]. Lastly, FA/HR factors are essential for fork recovery as supported by observations that individual or combined loss of BRCA2 and FANCD2 [20] or Rad51 impair replication fork-restart in human cells [21,22] (schematic in Figure 3A).

Therefore, we asked whether reduced levels of FA/HR factors in CagA-expressing cells might lead to replication stress-related DSBs arising as a consequence of impaired fork maintenance. Indeed, CagA expression increases the levels of pRPA (ser33), a marker of replication stress, while CagA-induced DSBs is exacerbated upon stalling fork progression induced by treatment with 0.2 mM HU for 8 h, (Figure S2B, C; Figure 3B, C).

Given the above evidence, replication fork dynamics such as speed and stability were studied in detail using the DNA fibre assay. Firstly, replication fork speed was measured by incorporating the nucleotide analogs chlorodeoxyuridine (CIdU) (green) for 40 min followed by iododeoxyuridine (IdU) (red) for 40 min (Figure 3D). Analysis of DNA fibres revealed that replication fork speed was reduced in CagA-expressing cells (median replication fork speed in CagA (−) 0.953 kb/min, CagA (+) 0.703 kb/min, *p* < 0.0001) (Figure 3D, right). The above data demonstrate an impairment of optimal replication speed in the presence of CagA. Furthermore, the reduced fork velocity was accompanied with an increased percentage of asymetric replication forks in CagA expressing cells (Figure 3E).

Since it is now established from different model systems that the loss of BRCA1, BRCA2, or Rad51 leads to single strand gaps at and behind the replication forks [16,23,24], we next questioned whether CagA-expressing cells accumulate single strand gaps during DNA replication using S1 nuclease assay according to Ke Cong et al., 2021 [23]. S1 nuclease treatment upon short CIdU incorporation resulted in a significant reduction in fibre lengths (Figure 3F) and this indicates the processing of replication fork intermediates during DNA replication.

To further confirm the nascent DNA processing in CagA expressing cells, replication fork stability was measured by challenging ongoing replication fork progression using hydroxyurea (HU). After incorporating CldU (green), cells were exposed to HU for 5 h and fibre length was measured (Figure 3G). Fibre analysis revealed significantly reduced CldU tract length in CagA-expressing cells indicating nascent DNA degradation (median length of CagA (−) 21.00 µM, CagA (+) 15.44 µM, *p* < 0.0001) (Figure 3G).

### 2.4. Poor Rad51 Chromatin Association, Increased MRE11 Nucleolytic Attack Triggers Replication Fork Instability and Stalled Replication Fork Formation in CagA Expressing Cells

**Figure 4:**
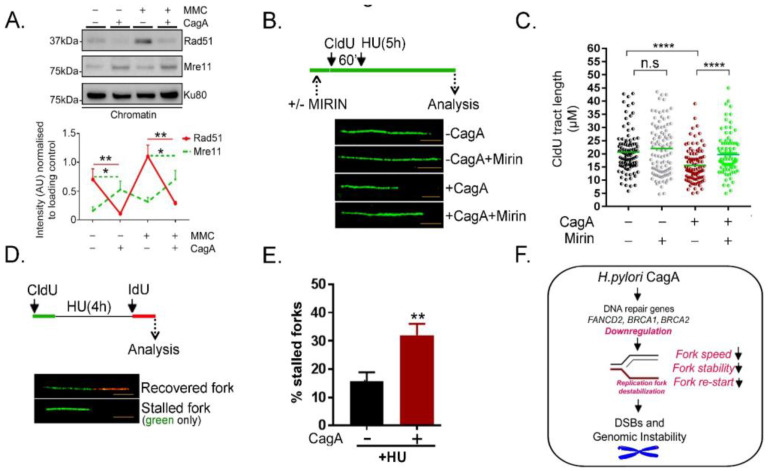
Poor Rad51 chromatin association in part contributes to compromised replication fork protection in CagA expressing cells (**A**). Dox inducible MKN28−CagA cells were either left untreated (−CagA) or subjected to doxycycline withdrawal (+CagA) for 48 h and left untreated or exposed to MMC (150 nM, 12 h). Immunoblot of chromatin fraction probed for Rad51 is shown. Right, quantification of the Western blot from three independent experiments were shown (**B,C**) Fork degradation in the presence or absence of Mirin**-**Fibre Assay Scheme: Cells were pre-treated with either DMSO alone or Mirin (50 μM for 30 min). Pulse labelling was then done with CldU for 60 min, following which cells they were exposed to HU (4mM) for 5 h. Replication fork degradation was calculated by measuring the median CIdU tract length, as shown in (k) *p =* <0.0001*,* n.s.—not significant. At least 100 fibres were scored for each experiment. The experiment was repeated thrice yielding similar results. (**D,E**) Fork stalling-Fibre assay Scheme: Cells were exposed to CldU for 40 min followed by HU for 4 h. Cells were then labelled with IdU for 60 min and “green-only” fibres were used to quantify percentage stalled forks. At least 100 fibres were scored for each experiment, n = 3. (**F**) Model explaining the defective replication fork recovery in CagA-expressing cells leading to genome instability. CagA expression results in downregulation of FANCD2, BRCA1, and BRCA2. As a consequence, the replication fork integrity in terms of replication speed, stability, and the ability to recover from damage is impaired resulting in double strand breaks and genomic instability. Asterisks represent significant differences. * *p* < 0.05; ** *p* < 0.01; **** *p* < 0.0001.

BRCA1/2 loss has been shown to reduce Rad51 chromatin association in both unperturbed and DNA damaging conditions [14,16]. Furthermore, BRCA1/2 and Rad51 have been shown to counteract MRE11 nuclease activity at forks to prevent nascent DNA degradation and single strand gap accumulation [14,16,17]. We next tested the level of Rad51 and MRE11 in the presence and absence of Mitomycin C. CagA induction and MMC treatment induced increased accumulation of DNA Damage evident by Gamma-H2AX intensity (Figure S2E). As speculated, Rad51 levels were reduced while MRE11 association was reciprocally increased on CagA-expressing chromatin both in the presence and absence of MMC, implying possible nuclease activity at forks. (Figure 4A).

Therefore, we next asked whether inhibition or inactivation of hyper MRE11 activity can prevent nascent DNA degradation in CagA-expressing cells. Strikingly, prior incubation with Mirin, an MRE11 specific small-molecule inhibitor, substantially rescued DNA fibre degradation and restored replication fork protection in CagA-expressing cells (*p* < 0.0001) (Figure 4B, C and Figure S2D). The above data demonstrate that HR/FA downregulation and the resultant impaired fork protection by Rad51 triggered heighten fork degradation in the presence of CagA.

Lastly, we measured the percentage of stalled DNA replication forks by blocking ongoing DNA replication with 4 mM HU for 5 h and we then measured the efficiency of DNA synthesis resumption. Here, CldU (green) was incorporated for 40 min, followed by a 2 mM HU treatment for 5h and subsequent IdU (red) incorporation for 60 min. Labelling of the second track (red) indicates efficient replication fork restart and green only tracts indicate stalled DNA replication forks (Figure 4D). Interestingly, the percentage of stalled forks (‘green only’ fibres) was significantly elevated in the presence of CagA (Figure 4E). The above data demonstrate substantial impairment in the re-start of stalled replication forks upon CagA expression. In addition to single strand gap formation, this might be an additional mechanism for DSB induction upon CagA expression. Taken together, the above results show that owing to the downregulation of vital FA/HR factors, CagA-expressing cells have defects in replication fork speed, fork stability and fork restart (Figure 4F). We conclude that fork destabilisation translates into toxic DSBs and generates genomic instability in CagA-expressing cells.

## 3. Discussion

Bacterial pathogens have evolved indigenous mechanisms to hijack host genome maintenance mechanisms and disrupt genomic integrity [26]. Here, we present an example of a bacterial virulence factor interfering with host replication fork stability apparatus to induce genomic instability. Using RNA Sequencing, SILAC-based proteome quantification, chromatin fractionation, DNA fibre assay, and PFGE, we report that the injection of the bacterial protein CagA by *H. pylori* results in (1) downregulation of key FA and HR factors, (2) host replication fork instability evident by reduced fork speed and single strand gap formation, and (3) replication-associated DSB formation within the host genome. We conclude that the combined deregulation of the aforementioned processes compromises genome stability in CagA-expressing cells. Overall, our data revealed the mechanism of *H. pylori* CagA mediated genome instability at the level of replication forks displaying features of BRCAness.

ROS generation and nucleotide imbalance has been linked to reduction in replication fork speed and generation of single strand gaps [18,24]. Since *H. pylori* infection also trigger ROS production [3], the combination of HR/FA factors downregulation and ROS production might fuel genome instability in CagA expressing *H. pylori* strains. Furthermore, the single strand gaps generated upon *H. pylori* CagA infection might become the substrate for error-prone DNA polymerases to initiate cancer causing mutations. Overall, these results might be useful for mitigating the genotoxic effects of *H.pylori*-infected precancerous conditions. Additionally, our unbiased transcriptomic and proteomic analysis following CagA expression constitutes a useful resource to interrogate novel functions of CagA.

How can membrane-associated CagA elicit transcriptional changes in DNA repair genes? Once CagA is delivered within the host cell, it interferes with several host signalling networks regulated by the Ras-ERK pathway or the WNT pathway [3]. Additionally, CagA is also known to activate the NFκB network which is known to regulate the expression of several DNA repair genes. CagA expression also modulates microRNA expression, DNA methylation, and histone methylation status of target gene promoters [25]. By using inhibitors against the above-mentioned pathways, we found that multiple pleiotropic mechanisms are involved in downregulating the expression of the 31 DNA repair gene set (Krishnan and Ito, unpublished). Secondly, although our replication-stress associated studies (Figure 2) are restricted to proliferating cells, it is well-known that CagA can induce senescence in a fraction of the population [13]. Saito et al. have previously demonstrated CagA induces senescence but at the same time converts into an oncogenic driver through p21 regulation [13]. Consistent to this we observed increased cells in G1 with a reduction in the number of S and G2 phase cells upon 48 h of constant CagA induction (Figure 1I.). It remains to be seen if CagA-dependent changes in DNA repair factors are influenced by senescence phenotype or conversely whether impaired DNA repair potentiates senescence in some cells. Lastly, in a recent study, CagA-mediated Par1-inhibition was shown to generate DSBs in primary gastric epithelial cells [26]. Given that Par1 is critically involved in *H. pylori-*dependent pathogenesis [27], it needs to be studied if DNA repair factor deregulation is related to impair Par1 function. In summary, further detailed mechanistic analyses are needed to identify the key nodes de-regulating the expression of DNA repair genes following CagA expression.

Intriguingly, although GC risk can be significantly ameliorated after *H. pylori* eradication, malignancy cannot be prevented in a subset of high-risk cohorts [28,29]. Accordingly, CagA was proposed to induce a sequel of genetic changes in the host genome that may promote carcinogenesis even after the original *H. pylori* infection has been cleared. For example, CagA expression elicits features of chromosomal instability (CIN) by inducing structural and numerical chromosomal anomalies [11], increases oxidative stress, and increases mutation rate through AID (activation-induced cytidine deaminase) activation [3]. Referred to as the “hit and run” model of pathogenicity [3], CagA-induced epigenetic alterations or genomic instability take over the immediate pro-oncogenic actions of CagA and give rise to the long-term detrimental effects. In this context, CagA-induced downregulation of FA/HR genes might itself be transient and last only as long as CagA is being actively delivered to the cell, while the downstream effects of FA/HR downregulation on single-strand gap generation and subsequent mutagenesis by error-prone polymerases might be undesirably permanent. We envisage that further understanding of CagA-dependent disruption of fork stability and DSB generation might unveil new strategies to reduce the genome-destabilizing effects of *H. pylori* infection.

## 4. Materials and Methods

### 4.1. Cell Lines, Drugs, and Antibodies

CagA-inducible MKN-28 cell line (WTA10), MKN-45, MKN-28, and AGS cell lines were used throughout the study. WTA10 is a MKN28-derived stable transfectant that inducibly expresses CagA based on the Tet-off system. WTA10 cells were cultured in RPMI 1640 medium supplemented with 10% fetal bovine serum (FBS), 10 mM HEPES, 0.1 mM non-essential amino acid, 1 mM sodium pyruvate, 2 mM L-glutamine, G418 (0.5 mg/mL), Hygromycin B (0.1 mg/mL), and Doxycycline (1 μg/mL). For induction of CagA, cells were washed thrice with PBS and cultured in the medium devoid of Doxycycline, as described in Figure S1A. MKN-28 and AGS were cultured in RPMI-1640 medium supplemented with 10% FBS.

For SILAC labelling, MKN-28 cells were cultured in RPMI 1640 (−Arg, −Lys) medium containing 10% dialysed fetal bovine serum (Thermo) supplemented with 84 mg/l ^13^C_6_^15^N_4_ l-arginine and 50 mg/l ^13^C_6_^15^N_2_ l-lysine (Cambridge Isotope) (“Heavy” medium) or the corresponding non-labelled amino acids (“Light” medium). Successful SILAC incorporation was verified by in-gel trypsin digestion and MS analysis (see below) of heavy input samples to ensure an incorporation rate of at least 98%.

Indicated concentrations of Hydroxyurea (HU) (Sigma Aldrich, #H8627), Formaldehyde (Sigma Aldrich, Singapore, #252549), and Mitomycin C (Sigma Aldrich, #M4287) were used in this study. The antibodies used for this work are as under γ-H2AX (1:1000, Millipore, clone JBW301, #05-636), Rad51 (1:1000, Abcam, #ab63801), KU80 (CST, #2180), BRCA2 (1:1000, Millipore # OP95), BRCA1 (1:1000, #sc-6954), FANCI (1:2000, Bethyl laboratories, A300-212A), FANCD2 (1:2000, Novus Biologicals, #NB 100-182), Phospho-Serine/Threonine ATM/ATR substrate antibody (CST, #2851), HA (1:1000, Cell Signalling, #37243), β-ACTIN (1:1000, Sigma, #A5316), Tubulin (1:1000, Sigma).

### 4.2. H. pylori Infection

*H. pylori* infection was performed as described earlier [26]. Briefly, isogenic wild-type strain of *H. pylori* (NCTC11637) and CagA-deleted version (ΔCagA) were cultured in Trypticase soy agar with 5% sheep blood (BD Biosciences) at 37 °C in a humidified and microaerophilic chamber to allow colony formation. Colonies were then inoculated into Brucella broth (Sigma-Aldrich) supplemented with 10% FBS for 24 h. Gastric cancer cells were infected using the cultured *H. pylori* at the indicated M.O.I. for 24 h.

### 4.3. RNA Extraction and Q-PCR

Total RNA was extracted according to the manufacturer’s instructions (RNAeasy mini kit, Qiagen). Briefly, cells were lysed in RLT buffer supplemented with β-mercaptoethanol. After total RNA extraction, equal amounts of DNase-treated RNA were reverse transcribed into cDNA using oligoDT primers using the superscript II reverse transcriptase (Invitrogen). Q-PCR was performed using gene specific primers and the SYBR green qPCR master mix (Biorad). Samples were run on the ABI Prism 7500 real time PCR system (Applied Biosystems). Data were normalised against the expression of *GAPDH* and fold changes were derived by averaging across replicates. Primers sequences are as provided below.

*ATRIP-*FP 5′ATGCACTGCGTGGAGGTCCTGCA3′;

RP 5′ GGTTTCCGCGGCACAGAGGTCATCCAG 3′

*BRCA1-*FP 5′CCACCCAATTGTGGTTGTGCAG3′

RP 5′GTAGGTGTCCAGCTCCTGGCA 3′

*BRCA2*-FP 5′GGCCGTACACTGCTCAAATC3′

RP 5′GCCATACAAAGTGATAAAGGAC 3′

*BRIP1*-FP 5′TATAAAGCTTACCCGTCACAGCTTGC3′

RP 5′ CTGTGGGACTCTCCAACAAACAATG 3′

*EME1-*FP 5′ GCAGTTGTGAATGCCTATCCCTCCCC 3′

RP 5′ CGAGCAAATTCTGGCGTTCTTTATCC 3′

*FANCD2*-FP 5′ GAGAGGCTTTCTGGCTGGG 3′

RP 5′ GGCTTTGCTCTTGGAGGCC 3′

*FANCE*-FP 5′ TCAGCCTCAGCAATGCTACTGT 3′

RP 5′ AAGGAGAGGATCCGTCCAAGA 3′

*FANCG*-FP 5′ TGTCCTCCTGACAGCATTTGC 3′

RP 5′ TGTCTGGGTTCCCTGTGATCA 3′

*FANCI*-FP 5′ GTGAACCTGATGCAGCACATG 3′

RP 5′ CCCATGCTCTGATGCAGTGC 3′

*PMS2*-FP 5′ AGATGTTTGCCTCCAGAGCCTGC 3′

RP 5′ CATGTGGGTGATCAGTTTCTTCATC 3′

*RPA3*-FP 5′ GCCACCATCTTGTGTACATCTTA 3′

RP 5′ GGGAAGTCATGGATAATTTTCACA 3′

*MSH2*-FP 5′ GCAAGGATATGATATCATGGAACCA 3′

RP 5′ GCATTTGTTTCACCTTGGACAGGAAC 3’

*MSH5*-FP 5′ CATCAAGCCTGTCAAGGATTTGCTA 3′

RP 5′ TCCAGGTTAGGATCTTCCAAATCCAGTTTC 3′

*MSH6*-FP 5′ GACATAGAAAAGCAAGAGAATTTGAG 3′

RP 5′ ACAGTTGACCTTTCACTAGCCAGG 3′

LIG1-FP 5′ CCGTGAAGACAAGCAGCCGGAGCAG 3′

RP 5′ CTCGCCTTGTTGGTTCTGAATCTGAC 3′

*PCNA*- FP 5′ GGAAATGGAAACATTAAATTGTCACAGA 3′

RP 5′ CTTTTGTAAAGAAGTTCAGGTACCTC 3′

*POLD1*-FP 5′ GTCGCCCATCCGGCTGGAGTTTGA 3′

RP 5′ GGCGTCGGGCCGGGAGGAGAAGA 3′

CHEK1-FP 5′ CCAGTGGATTTTCTAAGCACATTCAATCC 3′

RP 5′ AGACCTGTGCGGGGTTCTGGCTG 3′

*CHEK2*-FP 5′ GTTGGTAGTGGATCCAAAGGCACG 3′

RP 5′ CAGAAGATCTTGAAACTTTCTCTTCA 3′

*HLTF*-FP 5′ GTCCTTAAAAGCAGGTGGAGTTGGTTTGA 3′

RP 5′ CAGCATATTTTCTTCAACAGAGTCCTTTAC 3′

*MDC1*-FP 5′ GATTATTAGCTGCTGTGGAGGCACATAC 3′

RP 5′ GGAATGGAGCAATGAGGGAAGTCCTGA 3′

*MGMT*-FP 5′ TTCGGAGAAGTGATTTCTTACC 3′

RP 5′ CCGGAGTAGTTGCCCACGGCTCCG 3′

*NEIL3*-FP 5′ GGCAGTTTTATGCCTGTCCTCTACCTAGA 3′

RP 5′ TCATGGTGGAACGCTTGCCATGGTTGC 3′

*NHEJ1*-FP 5′ GCATTACAGTGCCAAGTGAGGGA 3′

RP 5′ TCATAAATTGTTCCAAGAAGGAAT 3′

*NTHL1*-FP 5′ CAAGTCCCCAGAGGAGACCCGCGCC 3′

RP 5′ CTGCTGGCCGAAGCCCACCAAGAGTCC 3′

*PARP1*-FP 5′ CGTTCCTCTTGGGACCGGGATTTC 3′

RP 5′ GTTTCAGCAGATACTTCAGATTTACC 3′

*POLQ*-FP 5′ GAGGAGGCTTCTTCATCCTTCA 3′

RP 5′ ATTTCACAGACAGTTTTACAGCAC 3′

*RAD51B*-FP 5′ CCGTCTGAGTAGACATCAGATCCTT 3′

RP 5′ CTCGATAACTCAGACCAGTCACCTTC 3′

*RAD51D*-FP 5′ CAGCACTCGGATTCTCCTGGACACC 3′

RP 5′GCACTCTGCTCTGAGGTCCCCCAGGTCCCAA 3′

*RAD54B*-FP5′GATTGTGTTACTCATGATCTGCTTGAC3′

RP 5′ GGTTTCAGGGAGTTAGATTTCTGGTG 3′

*RAD54L*-FP5′GACTTTGGATCTCTTTGAGAAGCTG3′

RP 5′ GAAGCGTTCTACAACCTTGGCTCGCTTC 3′

### 4.4. DNA Fibre Assay

Briefly, 10^5^ cells were pulse-labelled with 50 μM CIdU for 40 min, washed thrice with 1XPBS, and pulse-labelled with 250 μM IdU for 40 min with or without 4 mM Hydroxyurea (HU). After labelling, pulse-labelled cells were harvested with ice cold 1XPBS and 4 μL cell suspension were mixed with 6μL lysis buffer containing 200 mM Tris pH 7.5, 50 mM EDTA, and 0.5% SDS. The resultant mixture was layered on superfrost slides and pipetted vigorously. After 3 min of incubation, slides were tilted at 45° angle to allow cell suspension to flow to the end of the slide. Slides were dried and fixed with 3:1 methanol: Acetic acid overnight at 4 °C. Slides were rehydrated and treated with 2.5 M HCl for denaturation of DNA for 60 min. Next, slides were washed thrice with 1X PBS and incubated in blocking buffer (1%BSA, 1XPBS, and 0.1%Triton-X). CIdU was detected by incubating mouse anti-BrdU antibody (1:100) (Becton Dickinson, 347580) for 120 min. Then, slides were washed thrice with blocking buffer and incubated with AlexaFluor 488 conjugated goat anti mouse IgG (1:250). Then, IdU was detected by incubating rat anti-BrdU antibody (1:500) (Abcam #ab6326) for 120 min. Then, slides were washed thrice and incubated with AlexaFluor 594 conjugated goat anti mouse IgG (1:1000). Fibre images were acquired using the Olympus FluoView FV1000 Confocal microscope using the 60 × oil objective. For each experiment, at least 100 fibres were counted. CIdU and IdU tracts were measured using the ImageJ software (National Institute of Health). Replication fork speed in terms of kb/min was calculated as described previously [14].

### 4.5. Pulsed-Field Gel Electrophoresis

PFGE was performed as described previously [30]. Sub-confluent cultures of WTA10 cell line grown in the presence of absence of Doxycycline were treated with mentioned drugs and time duration later, cells were harvested by trypsinisation, and agarose plugs of 0.5–1.0 × 10^6^ cells were prepared with a CHEF disposable plug mold (BioRad) using ultrapure DNA grade agarose. Embedded cells within the plugs were incubated in lysis buffer (100 mM EDTA, 1% (*w*/*v*) sodium lauryl sarcosyne, 0.2% (*w*/*v*) sodium deoxycholate, 1 mg/mL proteinase K) at 37 °C for 24–36 h and then subjected to 4X (each 30′) washes in TE buffer (10 mM Tris-HCl (pH 8.0), 100 mM EDTA) before loading them onto a 1% pulse field certified agarose. Electrophoresis was performed for 24 h at 10 °C in 1% (*w*/*v*) agarose containing 0.5X Tris-borate with EDTA (TBE) using a CHEF-DR^®^ III Pulsed Field Electrophoresis Systems with the following parameters: voltage 6 V/cm; initial pulse and final pulse 5 s; Tm 14 °C, duration 24 h. Used electrophoresis conditions enable to resolve as high–*M*_W_ genomic DNA (more than several million base pairs (bp) remains in the well, whereas lower *M*_W_ DNA fragments (several Mbp to 500 kbp) migrate into the gel and are compacted into a single band. The gel was stained with ethidium bromide and analysed using ImageLab5.2 (BioRad). Band intensities were quantified using ImageQuant 5.2 software (GE Healthcare).

### 4.6. Mass Spectrometry Data Acquisition and Analysis

CagA was induced in SILAC labelled MKN-28 cells by the withdrawal of doxycycline for 72 h and cells were harvested. In the “forward” experiment, CagA was expressed in cells grown in the “heavy medium” by withdrawing doxycycline from the medium. While in the “reverse” experiment, CagA was expressed in cells grown in the “light medium”. Total cells extracts were prepared by re-suspending pellets in 3x packed cell volume of ice-cold suspension buffer (100 mM NaCl, 10 mM Tris, 1 mM EDTA) supplemented with complete protease inhibitors (Roche), HALT phosphatase inhibitor (Pierce), and 1 mM phenylmethylsulfonyl fluoride (PMSF). After incubation on ice for 5 min, an equal volume of 2X gel loading buffer (4% SDS, 200 mM DTT, 100 mM Tris, pH 6.8, 20% glycerol) was added and samples were boiled for 5 min at 95°C, sonicated, and spun down at 13,500 *g* at room temperature for 15 min. Protein concentrations were determined using the BCA protein assay reagent (Pierce). Equal amounts of CagA control and CagA-expressing extracts were mixed, boiled in 1X SDS loading buffer for 5 min, and 100 μg were run on a 12% Bis-Tris gel (NuPAGE, Thermo) for 30 min at 170 V in 1x MOPS buffer. The gel was fixed using the Colloidal Blue Staining Kit (Thermo) and each lane was divided into 4 equal fractions of different *M*_W_. For in-gel digestion, samples were destained in destaining buffer (25 mM ammonium bicarbonate; 50% ethanol) and reduced in 10 mM DTT for 1h at 56 °C followed by alkylation with 55 mM iodoacetamide (Sigma) for 45 min in the dark. Tryptic digest was performed in 50 mM ammonium bicarbonate buffer with 2 μg trypsin (Promega) at 37 °C overnight. Peptides were desalted on StageTips and analysed by nanoflow liquid chromatography on an EASY-nLC 1200 system coupled to a Q Exactive HF mass spectrometer (Thermo). Peptides were separated on a C18-reversed phase column (25 cm long, 75 μm inner diameter) packed in-house with ReproSil-Pur C18-QAQ 1.9 μm resin (Dr Maisch). The column was mounted on an Easy Flex Nano Source and temperature controlled by a column oven (Sonation) at 40 °C. A 215-min gradient from 2 to 40% acetonitrile in 0.5% formic acid at a flow of 225 nl/min was used. Spray voltage was set to 2.4 kV. The Q Exactive HF was operated with a TOP20 MS/MS spectra acquisition method per MS full scan. MS scans were conducted with 60,000 at a maximum injection time of 20 ms and MS/MS scans with 15,000 resolution at a maximum injection time of 50 ms. The raw files were processed with MaxQuant [31] version 1.5.2.8 with preset standard settings for SILAC labelled samples and the re-quantify option was activated. Carbamidomethylation was set as fixed modification while methionine oxidation and protein N-acetylation were considered as variable modifications. Search results were filtered with a false discovery rate of 0.01.

The mass spectrometry data is deposited to the ProteomeXchange Consortium via PRIDE [32] and the project accession number is PXD031168.

### 4.7. Chromatin Fractionation

Chromatin fractionation was performed as previously described [33]. Briefly, harvested cell pellets were resuspended in CSK buffer (10mM PIPES pH 6.8, 100 mM NaCl, 300 mM Sucrose, 1 mM EDTA, 1 mM EGTA, 1 mM MgCl_2_, and 0.5% Triton-X) and incubated at room temperature for 3 min. The resuspended lysate was spun at 1500× *g*, 4 °C for 5 min in swinging bucket rotor. Supernatant was collected as chromatin unbound extract. The chromatin pellet was washed twice with CSK buffer at 1500× *g*, 4 °C for 5 min. 2X packed cell volume of RIPA (20 mM Tris-HCl, (pH 8.0), 420 mM NaCl, 0.5% NP-40, 0.1 mM EDTA, 10% glycerol) was added for high-salt extraction of chromatin bound proteins by incubating samples for 30 min in ice. Finally, the mixture was centrifuged at 12,500× *g* for 15 min and the supernatant was Western blotted as chromatin fraction.

### 4.8. Immunofluorescence

Cells were fixed with 4% Paraformaldehyde for 15 min at room temperature. Following permeabilization with 0.5% Triton X-100 in PBS for 15 min, samples were blocked using 2% BSA, 5% FBS in 0.1% Triton X-100 for 30 min. Cells were incubated with antibodies diluted in 2% BSA in 0.1% Triton X-100 overnight at 4 °C. Alexa Fluor-conjugated secondary antibodies (1:1000) were added for 1 h at room temperature and coverslips were mounted with Prolong Gold Anti-fade (Invitrogen) containing DAPI. The Olympus FluoView FV1000 Confocal microscope was used for viewing images using the 40X oil objective. For quantitative imaging, cells were cultured on black-walled 96-well plates and immunofluorescence staining was performed as described above. The Operetta High-content imaging system (Perkin Elmer) was used to quantify intensity of nuclear fluorescence signals.

### 4.9. Cell Cycle Analysis (FACS)

The cells are harvested, washed with ice-cold 1XPBS, fixed with 75% ethanol, and incubated for 30 min on ice. The pellet of 1 × 10^6^ cells was re-suspended in 1 mL staining solution (50 µg/mL propidium iodide + 1mg/mL RNase) and incubated at 4 °C overnight. Cell cycle acquisition was performed on FACS Calibur BD platform and analysis was done in flowjo10.

### 4.10. RNA-Sequencing and Raw Data Processing

Samples were harvested and total RNA was extracted according to the manufacturer’s instructions (QIAGEN RNeasy mini kit). Samples were processed further for RNA-Seq analysis, as described previously [33]. The data have been deposited under the gene expression omnibus (GEO) accession number GSE132356.

## Data Availability

The data presented in this study are available on request from the corresponding authors.

## Supplementary Materials

The following are available online at https://www.mdpi.com/article/10.3390/ijms23031661/s1.
