# Supplementary material for: The H. pylori CagA Oncoprotein Induces DNA Double Strand Breaks through Fanconi Anemia Pathway Downregulation and Replication Fork Collapse
Source: Int J Mol Sci. 2022 Jan 31;23(3):1661. doi: 10.3390/ijms23031661 (PMC8836099; doi:10.3390/ijms23031661)
Supplement: Supplementary file 1 [file ijms-23-01661-s001.zip › Kolinjivadi et al., Supplement Data Final (1).pdf]

## LEGENDS (SUPPLEMENTARY FIGURES)

### Figure.S1. Downregulation of FA repair factors by CagA

**A.** Schematic depicting the induction of CagA in Dox-inducible MKN28 cells.

**B-D.** Dox-inducible MKN28-CagA cells were either left untreated (-CagA) or subjected to doxycycline withdrawal (+CagA) for 48 hours. Quantification of nuclear fluorescence of indicated proteins (p-ATM-ser1981, p-SQ/TQ and p-CHK2-thr68) in the presence or absence of CagA using the operetta high-high content imaging system (methods). P***=$p$<0.001

**E.** (i) Dox-inducible MKN28-CagA cells were left untreated (-CagA) or subjected to doxycycline withdrawal (+CagA) for 72 hours followed by RNA-seq analysis. Gene Ontology analysis of Differentially Expressed Genes (DEGs) was done using the DAVID GO tool.

(ii) Enrichment plot of curated DNA repair gene list (172 genes) from Geneset Enrichment Analysis (GSEA). Enrichment score (*y*-axis) was plotted against rank position of the 172 DNA repair genes (*x*-axis).

**F.** Dox-inducible MKN28-CagA cells were either left untreated (-CagA) or subjected to doxycycline withdrawal (+CagA) for 72 hours and RNA was subjected to Q-PCR analysis of the indicated genes. All graphs show mean±S.D. Asterisks represent significant differences. P*=$p$<0.05; P**=$p$<0.01, P***=$p$<0.001

**G.** AGS cells were infected with wild-type *H.pylori* or *H.pylori* (ΔCagA) at the M.O.I of 100 for 24 hours and subjected to Q-PCR analysis

### Figure.S2. Replication Stress induction upon CagA expression

**A.** A global view of the proteins that are differentially expressed upon CagA induction (72 hours). The cut-offs for significant differential expression were set to log2(fold change) >1 across the forward (X-axis) and reverse (Y-axis) SILAC-based proteomic quantifications. Red and blue circles depict proteins upregulated or downregulated by at least 2-fold upon CagA expression, respectively.

**B.** Dox-inducible MKN28-CagA cells were either left untreated (-CagA) or subjected to doxycycline withdrawal (+CagA) for 48 hours and immunofluorescence analysis was performed with the p-RPA (ser33) antibody as a marker of replication stress. Scale bar=50μm

**C.** For the experiment indicated in **B**, quantification of nuclear fluorescence of pRPA-ser33 is shown. P***=$p$<0.001

**D.** For the experiment shown in Main **Figure.4B-C**, frequency of CldU track length distribution comparison between –CagA and +CagA (left) and between –CagA and +CagA in the presence of Mirin (right) are shown.

**F.** Dox inducible MKN28−CagA cells were either left untreated (−CagA) or subjected to doxycycline withdrawal (+CagA) for 48 h and left untreated or exposed to MMC (150 nM, 12 h). Quantification of nuclear Gamma-H2AX intensity is shown.

# Figure.S1

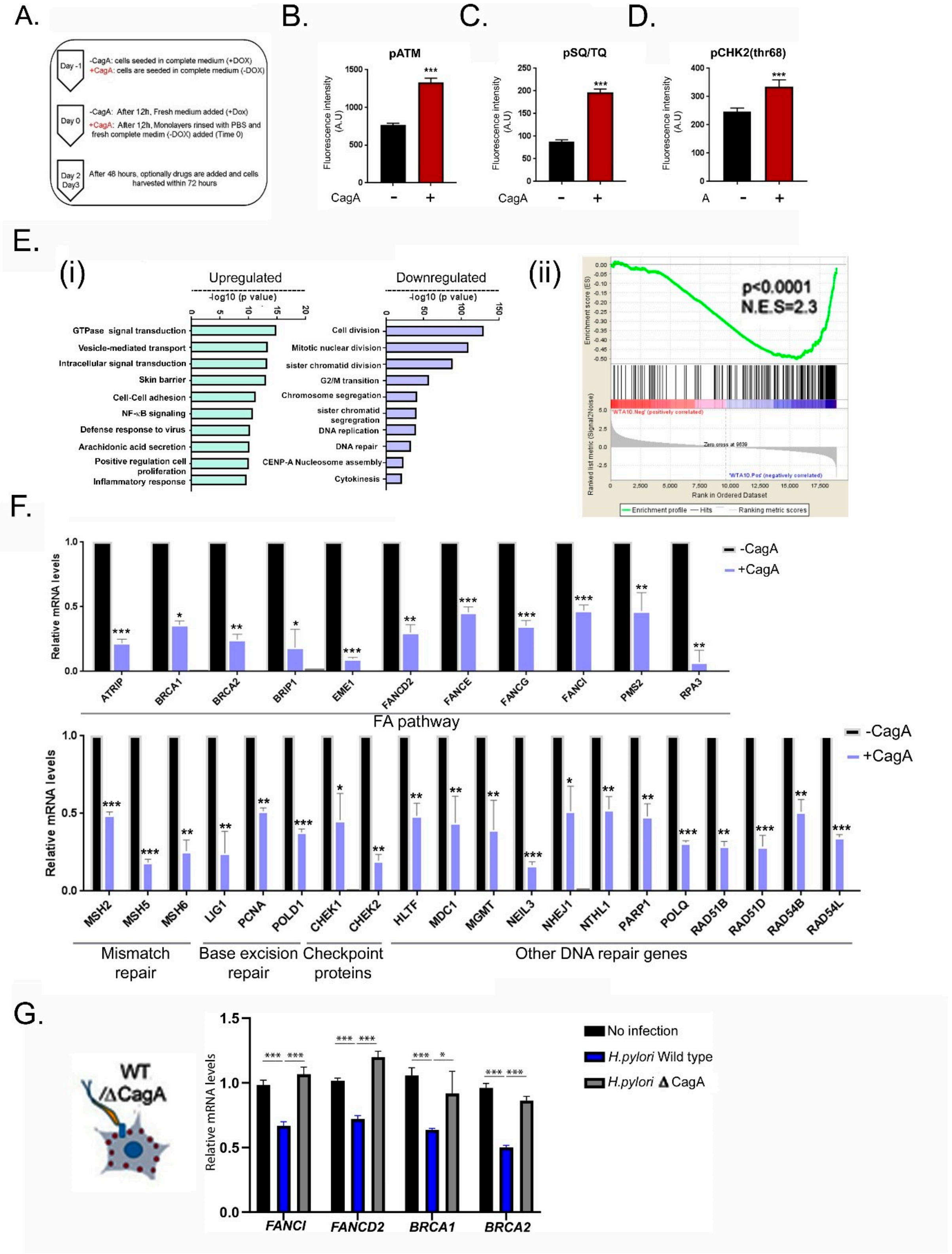

A.
Day -1
-CagA: cells seeded in complete medium (+DOX)
+CagA: cells are seeded in complete medium (-DOX)
Day 0
-CagA: After 12h, Fresh medium added (+Dox)
+CagA: After 12h, Monolayers rinsed with PBS and fresh complete medim (-DOX) added (Time 0)
Day 2 Day3
After 48 hours, optionally drugs are added and cells harvested within 72 hours
B.
pATM
C.
pSQ/TQ
D.
pCHK2(thr68)
Fluorescence intensity (A.U)
CagA
E.
(i)
Upregulated
-log10 (p value)
GTPase signal transduction
Vesicle-mediated transport
Intracellular signal transduction
Skin barrier
Cell-Cell adhesion
NF-κB signaling
Defense response to virus
Arachidonic acid secretion
Positive regulation cell proliferation
Inflammatory response
Downregulated
Cell division
Mitotic nuclear division
sister chromatid division
G2/M transition
Chromosome segregation
sister chromatid segregation
DNA replication
DNA repair
CENP-A Nucleosome assembly
Cytokinesis
(ii)
p<0.0001
N.E.S=2.3
Enrichment score (ES)
Ranked list metric (Signal2Noise)
Rank in Ordered Dataset
Enrichment profile
Hits
Ranking metric scores
F.
Relative mRNA levels
-CagA
+CagA
ATRIP
BRCA1
BRCA2
BRIP1
EME1
FANCD2
FANCE
FANCG
FANCI
PMS2
RPA3
FA pathway
MSH2
MSH5
MSH6
LIG1
PCNA
POLD1
CHEK1
CHEK2
HLTF
MDC1
MGMT
NEIL3
NHEJ1
NTHL1
PARP1
POLQ
RAD51B
RAD51D
RAD54B
RAD54L
Mismatch repair
Base excision repair
Checkpoint proteins
Other DNA repair genes
G.
WT /ΔCagA
Relative mRNA levels
No infection
H.pylori Wild type
H.pylori Δ CagA
FANCI
FANCD2
BRCA1
BRCA2

**Figure.S2**

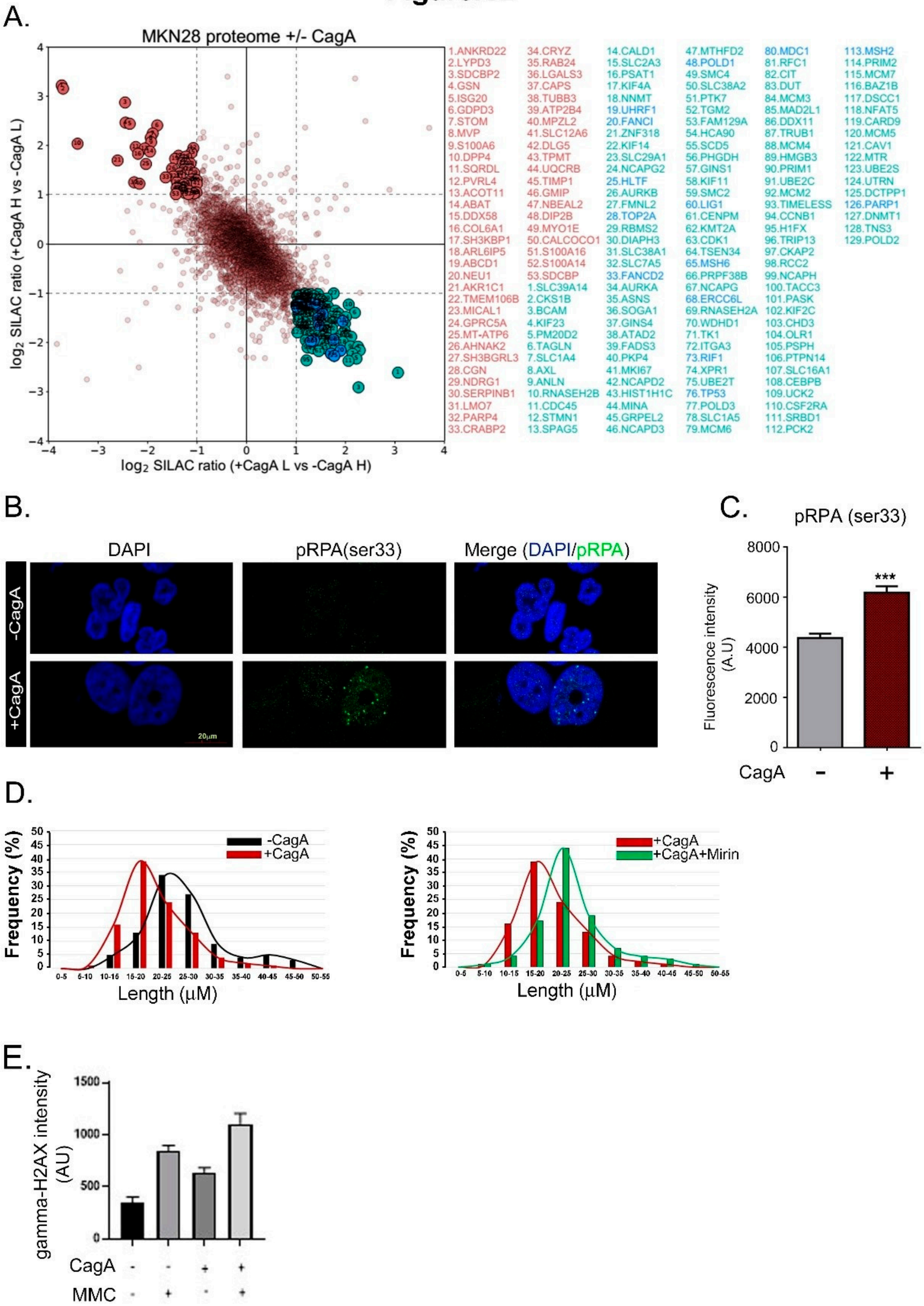
A.
MKN28 proteome +/- CagA
log2 SILAC ratio (+CagA H vs -CagA L)
log2 SILAC ratio (+CagA L vs -CagA H)
1.ANKRD22 2.LYPD3 3.SDCBP2 4.GSN 5.ISG20 6.GDPD3 7.STOM 8.MVP 9.S100A6 10.DPP4 11.SQRDL 12.PVRL4 13.ACOT11 14.ABAT 15.DDX58 16.COL6A1 17.SH3KBP1 18.ARL6IP5 19.ABCD1 20.NEU1 21.AKR1C1 22.TMEM106B 23.MICAL1 24.GPRC5A 25.MT-ATP6 26.AHNAK2 27.SH3BGRL3 28.CGN 29.NDRG1 30.SERPINB1 31.LMO7 32.PARP4 33.CRABP2
34.CRYZ 35.RAB24 36.LGALS3 37.CAPS 38.TUBB3 39.ATP2B4 40.MPZL2 41.SLC12A6 42.DLG5 43.TPMT 44.UQCRB 45.TIMP1 46.GMIP 47.NBEAL2 48.DIP2B 49.MYO1E 50.CALCOCO1 51.S100A16 52.S100A14 53.SDCBP
1.SLC39A14 2.CKS1B 3.BCAM 4.KIF23 5.PM20D2 6.TAGLN 7.SLC1A4 8.AXL 9.ANLN 10.RNASEH2B 11.CDC45 12.STMN1 13.SPAG5
14.CALD1 15.SLC2A3 16.PSAT1 17.KIF4A 18.NNMT 19.UHRF1 20.FANCI 21.ZNF318 22.KIF14 23.SLC29A1 24.NCAPG2 25.HLTF 26.AURKB 27.FMNL2 28.TOP2A 29.RBMS2 30.DIAPH3 31.SLC38A1 32.SLC7A5 33.FANCD2 34.AURKA 35.ASNS 36.SOGA1 37.GINS4 38.ATAD2 39.FADS3 40.PKP4 41.MKI67 42.NCAPD2 43.HIST1H1C 44.MINA 45.GRPEL2 46.NCAPD3
47.MTHFD2 48.POLD1 49.SMC4 50.SLC38A2 51.PTK7 52.TGM2 53.FAM129A 54.HCA90 55.SCD5 56.PHGDH 57.GINS1 58.KIF11 59.SMC2 60.LIG1 61.CENPM 62.KMT2A 63.CDK1 64.TSEN34 65.MSH6 66.PRPF38B 67.NCAPG 68.ERCC6L 69.RNASEH2A 70.WDHD1 71.TK1 72.ITGA3 73.RIF1 74.XPR1 75.UBE2T 76.TP53 77.POLD3 78.SLC1A5 79.MCM6
80.MDC1 81.RFC1 82.CIT 83.DUT 84.MCM3 85.MAD2L1 86.DDX11 87.TRUB1 88.MCM4 89.HMGB3 90.PRIM1 91.UBE2C 92.MCM2 93.TIMELESS 94.CCNB1 95.H1FX 96.TRIP13 97.CKAP2 98.RCC2 99.NCAPH 100.TACC3 101.PASK 102.KIF2C 103.CHD3 104.OLR1 105.PSPH 106.PTPN14 107.SLC16A1 108.CEBPB 109.UCK2 110.CSF2RA 111.SRBD1 112.PCK2
113.MSH2 114.PRIM2 115.MCM7 116.BAZ1B 117.DSCC1 118.NFAT5 119.CARD9 120.MCM5 121.CAV1 122.MTR 123.UBE2S 124.UTRN 125.DCTPP1 126.PARP1 127.DNMT1 128.TNS3 129.POLD2
B.
DAPI
pRPA(ser33)
Merge (DAPI/pRPA)
-CagA
+CagA
20μm
C.
pRPA (ser33)
Fluorescence intensity (A.U)
***
CagA
D.
Frequency (%)
-CagA
+CagA
Length (μM)
+CagA
+CagA+Mirin
E.
gamma-H2AX intensity (AU)
CagA
MMC
